# Meta QTL analysis for dissecting abiotic stress tolerance in chickpea

**DOI:** 10.1186/s12864-024-10336-9

**Published:** 2024-05-02

**Authors:** Sourav Panigrahi, Upendra Kumar, Sonu Swami, Yogita Singh, Priyanka Balyan, Krishna Pal singh, Om Parkash Dhankher, Rajeev K Varshney, Manish Roorkiwal, Khaled MA Amiri, Reyazul Rouf Mir

**Affiliations:** 1 Department of Molecular Biology & Biotechnology, College of Biotechnology, CCS Haryana Agricultural University, Hisar, 125004 India; 2 Department of Plant Science, Mahatma Jyotiba Phule Rohilkhand University, Bareilly, 243001 India; 3 Department of Botany & Plant Physiology, College of Basic Sciences & Humanities, CCS Haryana Agricultural University, Hisar, 125004 India; 4 Department of Botany, Deva Nagri P.G. College, CCS University, Meerut, 245206 India; 5 Biophysics Unit, College of Basic Sciences & Humanities, GB Pant University of Agriculture & Technology, Pantnagar, 263145 India; 6 Vice-Chancellor’s Secretariat, Mahatma Jyotiba Phule Rohilkhand University, Bareilly, 243001 India; 7 Stockbridge School of Agriculture, University of Massachusetts, Amherst, USA; 8 Centre for Crop & Food Innovation, State Agricultural Biotechnology Centre, Food Futures Institute, Murdoch University, Murdoch, WA Australia; 9 Khalifa Center for Genetic Engineering and Biotechnology, United Arab Emirates University, Al-Ain, United Arab Emirates; 10 Department of Biology, College of Science, United Arab Emirates University, Al-Ain, United Arab Emirates; 11 Division of Genetics and Plant Breeding, Sher-e-Kashmir University of Agricultural Sciences and Technology of Kashmir (SKUAST-Kashmir), Srinagar, J&K India

**Keywords:** Meta-analysis, Biomercator, Consensus map, Marker density, Explained phenotypic variance, GWAS

## Abstract

**Background:**

Chickpea is prone to many abiotic stresses such as heat, drought, salinity, etc. which cause severe loss in yield. Tolerance towards these stresses is quantitative in nature and many studies have been done to map the loci influencing these traits in different populations using different markers. This study is an attempt to meta-analyse those reported loci projected over a high-density consensus map to provide a more accurate information on the regions influencing heat, drought, cold and salinity tolerance in chickpea.

**Results:**

A meta-analysis of QTL reported to be responsible for tolerance to drought, heat, cold and salinity stress tolerance in chickpeas was done. A total of 1512 QTL responsible for the concerned abiotic stress tolerance were collected from literature, of which 1189 were projected on a chickpea consensus genetic map. The QTL meta-analysis predicted 59 MQTL spread over all 8 chromosomes, responsible for these 4 kinds of abiotic stress tolerance in chickpea. The physical locations of 23 MQTL were validated by various marker-trait associations and genome-wide association studies. Out of these reported MQTL, CaMQAST1.1, CaMQAST4.1, CaMQAST4.4, CaMQAST7.8, and CaMQAST8.2 were suggested to be useful for different breeding approaches as they were responsible for high per cent variance explained (PVE), had small intervals and encompassed a large number of originally reported QTL. Many putative candidate genes that might be responsible for directly or indirectly conferring abiotic stress tolerance were identified in the region covered by 4 major MQTL- CaMQAST1.1, CaMQAST4.4, CaMQAST7.7, and CaMQAST6.4, such as heat shock proteins, auxin and gibberellin response factors, etc.

**Conclusion:**

The results of this study should be useful for the breeders and researchers to develop new chickpea varieties which are tolerant to drought, heat, cold, and salinity stresses.

**Supplementary Information:**

The online version contains supplementary material available at 10.1186/s12864-024-10336-9.

## Introduction

Chickpea (*Cicer arietinum* L.), a protein rich legume crop, is cultivated in arid and semi-arid regions worldwide [1]. It has a global production of 15.08 million tons and an average productivity of 1016 kg per hectare [2–4]. However, abiotic stresses like drought, salinity, and heat/cold stress [5, 6], as well as rare but important ones like waterlogging and metal toxicity, cause significant yield loss. This has led to increased stress-mitigating concerns in chickpea-producing countries [5].

Chickpea genotypes exhibit varying thermo-tolerance i.e., up to 45 ˚C, during seed germination [7], with optimum temperatures ranging from 10 °C to 33 °C [8, 9]. High temperatures can lead to reduced pollen viability, pod abortion, and low seed filling [10–12]. Drought is another abiotic stress that negatively impacts chickpea yield [13, 14], causing membrane integrity loss and cell damage [15–19]. To ensure nutritional security for 2 billion people, chickpea varieties must have beneficial traits like nutritional quality and stress resistance [20]. Genetic enhancement based on known mechanisms and quantitative trait loci (QTL) is necessary to target these traits [1, 21]. QTL linked to plant height [22], flowering time [23], seed size/weight [24, 25] have also been identified. Abiotic stress tolerance is also influenced by genetic parameters and environmental interactions [1]. Similar approaches have been used to improve nutritional quality in other cereal crops like wheat [26, 27] and rice [28, 29].

Heat stress negatively impacts crop growth [30], particularly in cold season crops like chickpea [31, 32]. Chickpeas are most vulnerable to heat stress during pre-anthesis and anthesis [33]. It causes damage to chlorophyll [34–36], respiration [37], and membrane stability [38]. To measure heat stress tolerance, indices like cell membrane stability [39, 40], lipid composition, pollen heat shock proteins, and osmoregulator content have been studied [41–43]. Cold stress disrupts membranes [37, 44], hampers pollen germination, and affects photosynthesis [45, 46] and electron transport [47]. The stress-tolerant chickpea lines are evaluated [48], using various indices like drought response index (DRI), stress tolerance index (STI), tolerance (TOL), mean productivity (MP), geometric mean productivity (GMP) and Osmotic adjustment (OA) which are effective indicators of stress tolerance [49–53].

Chickpea production is also negatively impacted by salinity in arid and semi-arid regions [54, 55]. Salinity stress reduces water potential [56, 57], creates ions imbalances [58, 59], causes toxicity [60] and negatively affects nodulation, nodule size and N_2_ fixation [61–63]. Physiological parameters like stomatal conductance, evapotranspiration, leaf area, and other parameters like yield can determine tolerance against salinity [64]. Early maturity, higher pre-dawn water potential, osmotic adjustment, and branch retention can also provide tolerance [65].

Abiotic stress tolerance, such as drought tolerance is a complex attribute controlled by major-effect QTL and numerous small effect QTL [4]. QTL analysis is a valuable tool for identifying genomic regions controlling quantitative traits [66, 67]. Molecular markers and statistical methods have been developed to identify QTL, which contribute to abiotic stress tolerance in chickpea [68]. QTL are chromosomal locations of complex traits in chickpea, contributing to abiotic stress tolerance [69, 70], with positions on linkage groups, confidence intervals, R2/PVE (Percentage variance explained) and LOD (Logarithm of Odds) scores.

Meta-analysis is a study that combines data from multiple sources to confirm QTL’ location on the species’ genome. It aims to identify QTL hotspots over a consensus genetic map [71]. Studies have identified MQTL in wheat, rice, garden pea, pigeon pea, and soybean which address the various traits and constraints affecting them [68, 72–76], but chickpea has not yet been included. This study presents a meta-analysis of QTL governing abiotic stress tolerance in chickpea, providing breeders with more accurate information for enhancing yield and abiotic stress tolerance.

## Results

### Distribution of QTL on chickpea chromosomes

**Fig. 1:**
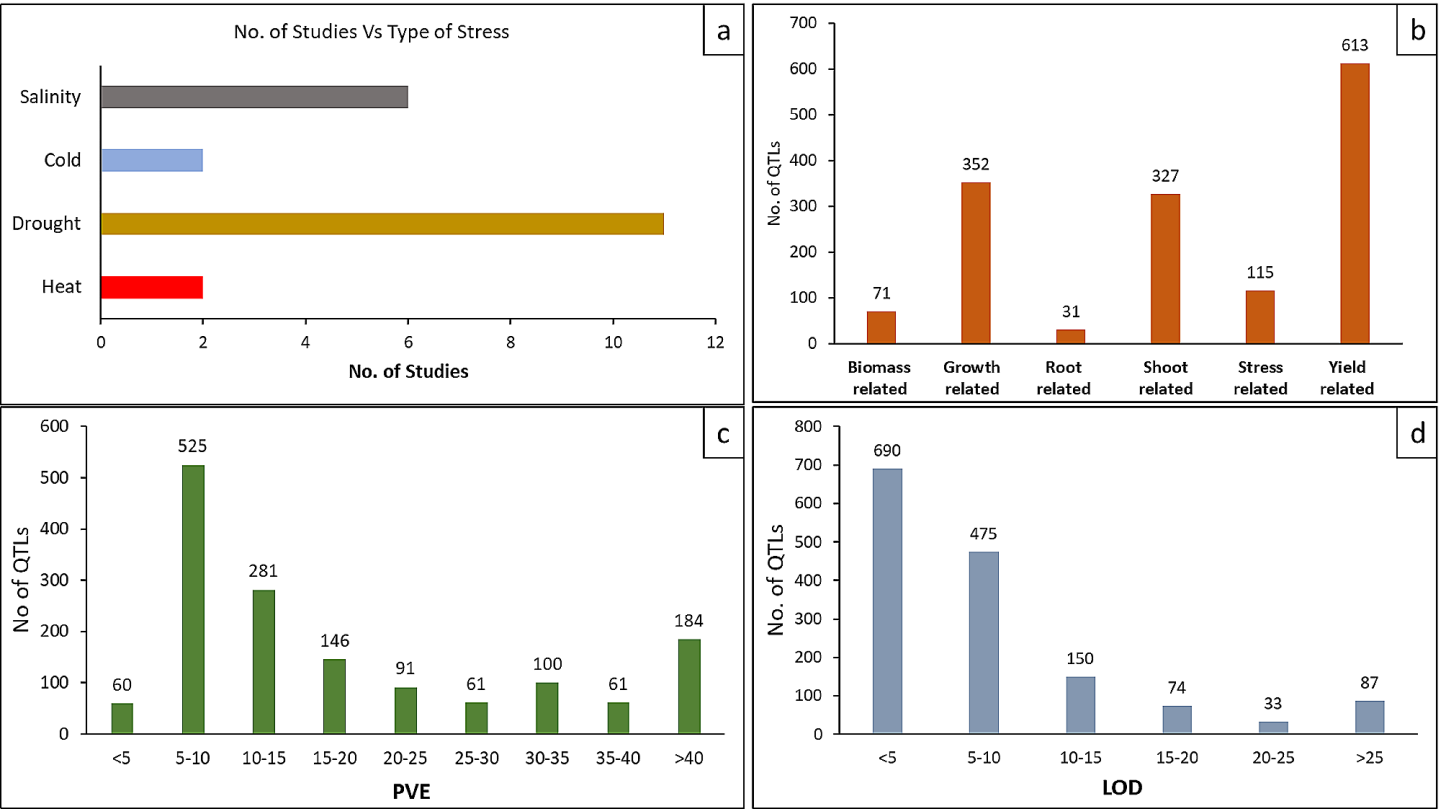
Characteristics of QTL related to abiotic stress tolerance as obtained in previous studies. (**a**) Number of studies according to the type of stress discussed, classification of QTL according to type of trait (**b**), PVE (**c**) and LOD (**d**)

**Fig. 2:**
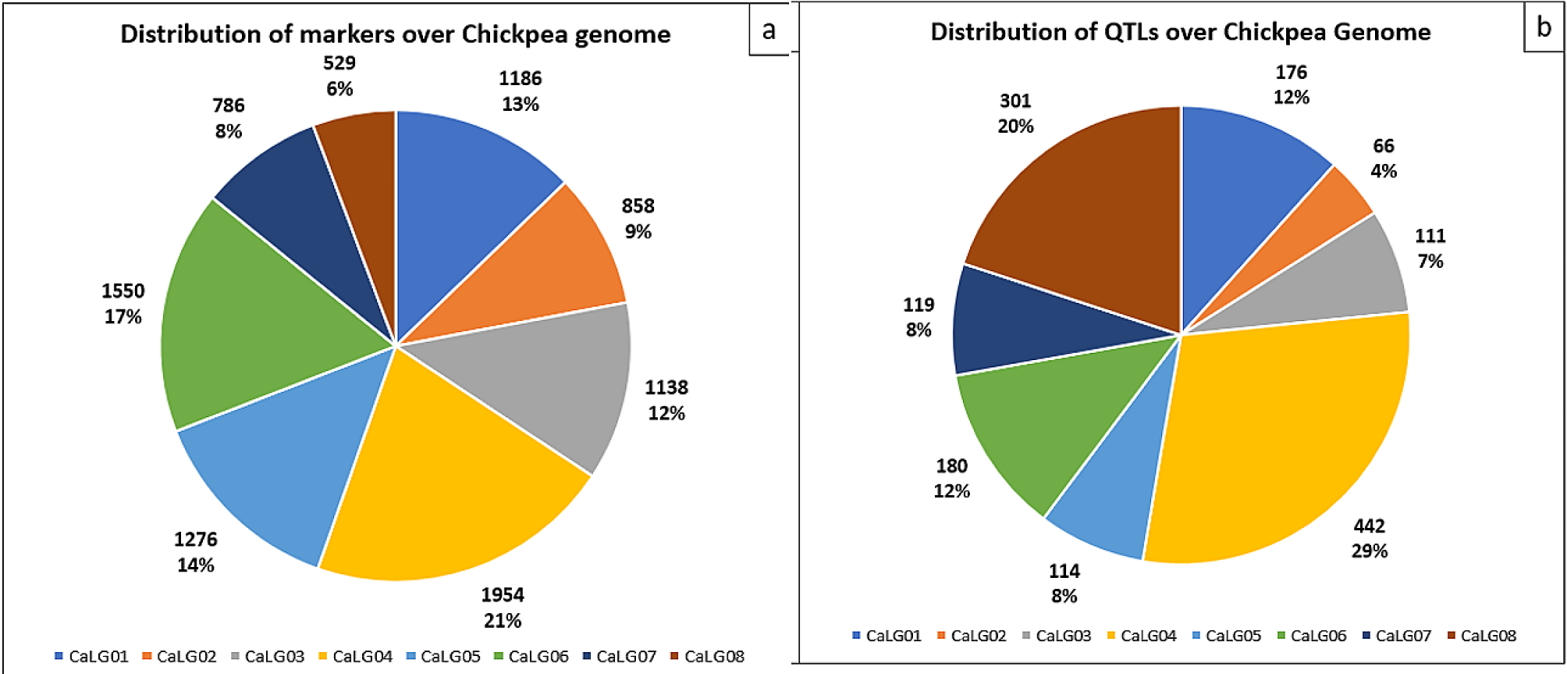
Distribution of markers (**a**) and QTL related to abiotic stress tolerance (**b**) in Chickpea genome

Twenty-one studies conducted during the period 2007–2022 were collected and characterized as they provided all the necessary data regarding the QTL taken for meta-analysis. These studies reported 1512 QTL which were directly or indirectly involved in abiotic stress tolerance in chickpeas such as drought, heat, and salinity (Fig. 1a), which were then listed with detailed information including flanking markers, location in the genome, LOD scores and R^2^ values for each QTL (Supplementary Table 1). These studies involved populations of different sizes and types. Although most of the studies involved RIL populations, Rehman et al. [77] and Jha et al. [104] used the F_2_ population, and Thudi et al. [78] used the MAGIC F_6_ population for QTL analysis. The population size varied from 126 to 1200. Different methods were employed to identify the QTL presented in the studies. Among the collected QTL most were located on chromosome 4 followed by chromosome 8. The least number of QTL involved in abiotic stress tolerance were present on Chromosome 2 (Fig. 2b). The number of QTL per trait varied from 1 for chlorophyll content, vernalization response, and sodium to 226 for the yield-related trait- ‘100 seed weight’ (Table 1; Fig. 1b). The various parameters for the collected QTL ranged from 2 to 54.9 concerning LOD scores (Fig. 1d) and 2 to 76.715 for the variance explained (PVE) by the QTL for their respective traits. A maximum number of QTL were observed to have PVE between 5 and 10% while a few of the QTL were observed to play a major role in abiotic stress tolerance having PVE > 25% (Fig. 1c).

**Table 1:** Distribution of the number of QTL identified for 64 traits/indices

Trait	CaLG01	CaLG02	CaLG03	CaLG04	CaLG05	CaLG06	CaLG07	CaLG08	Total
% Pod setting (%PS)	3	0	0	4	5	4	4	1	21
100-Seed weight (100SW)	51	2	10	120	8	17	6	12	226
3-D leaf area (3DL)	0	0	0	7	0	0	0	1	8
3-D leaf area growth rate (3DLG)	0	0	0	2	0	1	0	0	3
Aboveground dry matter (ADM)	0	0	0	0	3	0	0	0	3
Absolute growth rate at 30 DAS (AGR30)	1	0	0	1	0	0	0	0	2
Absolute growth rate at 34 DAS (AGR34)	1	0	0	1	0	0	0	0	2
Canopy temperature-Air temperature (Tc–Ta)	1	0	1	1	0	3	0	0	6
Chloride (Cl-)	0	0	0	1	1	0	0	0	2
Chlorophyll content (CC)	0	0	0	0	0	1	0	0	1
Cold tolerance (CT)	1	0	1	0	0	0	0	1	3
Days from flowering to maturity (DFM)	1	0	1	0	0	0	1	0	3
Days to 100% flowering (DTF)	0	1	0	0	4	1	0	0	6
Days to flowering (DF)	12	10	23	11	18	13	4	78	169
Days to flowering initiation (DFI)	0	1	0	3	0	2	0	0	6
Days to germination (DG)	0	1	0	0	0	1	1	0	3
Days to maturity (DM)	10	12	12	15	18	17	7	75	166
Delta carbon ratio (Δ13c)	0	0	0	6	0	0	0	0	6
Drought susceptibility indices (DSI)	1	0	1	3	0	0	3	3	11
Drought tolerance indices (DTI)	9	1	0	3	1	1	3	2	20
Drought tolerance score (DTS)	1	0	1	0	0	0	1	1	4
Evotranspiration (eT)	0	0	0	4	0	0	0	0	4
Evotranspiration rate (eTR)	0	0	3	6	0	0	1	0	10
Harvest index (HI)	13	4	12	19	7	16	5	31	107
Leaf area index (LAI)	2	0	0	10	0	0	0	2	14
Membrane permeability index (MPI)	0	0	0	0	0	3	2	0	5
Membrane stability index (MSI)	0	1	0	0	0	1	1	0	3
Necrosis (N)	0	0	1	2	1	0	0	0	4
Number of filled pod (NFP)	0	3	0	3	4	3	4	0	17
Number of pods/plant (NP/P)	0	1	1	25	0	5	2	6	40
Number of primary branches (NPB)	2	1	2	0	0	2	1	5	13
Number of secondary branches (NSB)	2	0	0	1	0	0	0	1	4
Number of Seed / Pod (NS/P)	0	0	1	5	1	0	1	1	9
Plant height (PHT)	7	0	14	64	9	33	17	41	185
Plant height growth rate (PHTG)	0	0	0	14	0	2	2	2	20
Plant stand (PS)	1	5	0	0	1	1	0	1	9
Plant vigor score (PBS)	1	0	2	3	0	0	0	1	7
Plant width (PWD)	0	1	1	1	1	0	0	2	6
Projected leaf area (PLA)	0	0	0	8	1	0	0	1	10
R-3D/Projected leaf area (R-3D/PLA)	4	0	0	10	0	8	12	1	35
Relative leaf water content (%) (RLWC)	0	1	0	3	1	1	1	0	7
Root dry weight (RDW)	1	0	2	0	0	0	1	2	6
Root length density (RLD)	0	0	0	7	2	1	1	0	11
Root surface area (RSA)	0	0	1	2	1	3	0	1	8
Root volume (RV)	0	0	0	0	0	1	1	1	3
Rooting depth (RDp)	0	0	1	0	1	0	0	1	3
R-T ratio (RTR)	1	0	1	11	1	1	1	2	18
Seed number (SN)	1	2	0	7	3	12	9	1	35
Seed size (SS)	1	0	0	1	0	0	0	0	2
Seed weight (SW)	3	2	6	3	2	1	2	0	19
Seedling Biomass (SBM)	1	0	0	1	0	0	0	0	2
Shoot dry weight (SDW)	0	0	0	3	0	1	0	0	4
Sodium (Na)	0	0	0	0	1	0	0	0	1
Specific leaf area (SLA)	0	0	0	3	0	2	0	0	5
Specific leaf weight (SLW)	0	0	0	2	0	0	0	1	3
Stomatal conductance (SC)	0	0	1	0	0	0	2	0	3
Total Biomass (BM)	9	5	6	9	5	3	8	13	58
Total pod number (TPN)	0	0	0	0	0	0	3	0	3
Transpiration (T)	0	0	0	4	1	1	0	1	7
Transpiration rate (TR)	0	0	2	2	0	0	2	0	6
Vernalization response (VR)	0	0	1	0	0	0	0	0	1
Visual score on podding behavior (VS)	8	2	0	0	3	3	1	3	20
Water use efficiency (WUE)	2	0	0	3	0	0	0	0	5
Yield / Plant (YLD)	25	12	6	22	14	15	9	6	109

### Consensus map and QTL projection

**Fig. 3:**
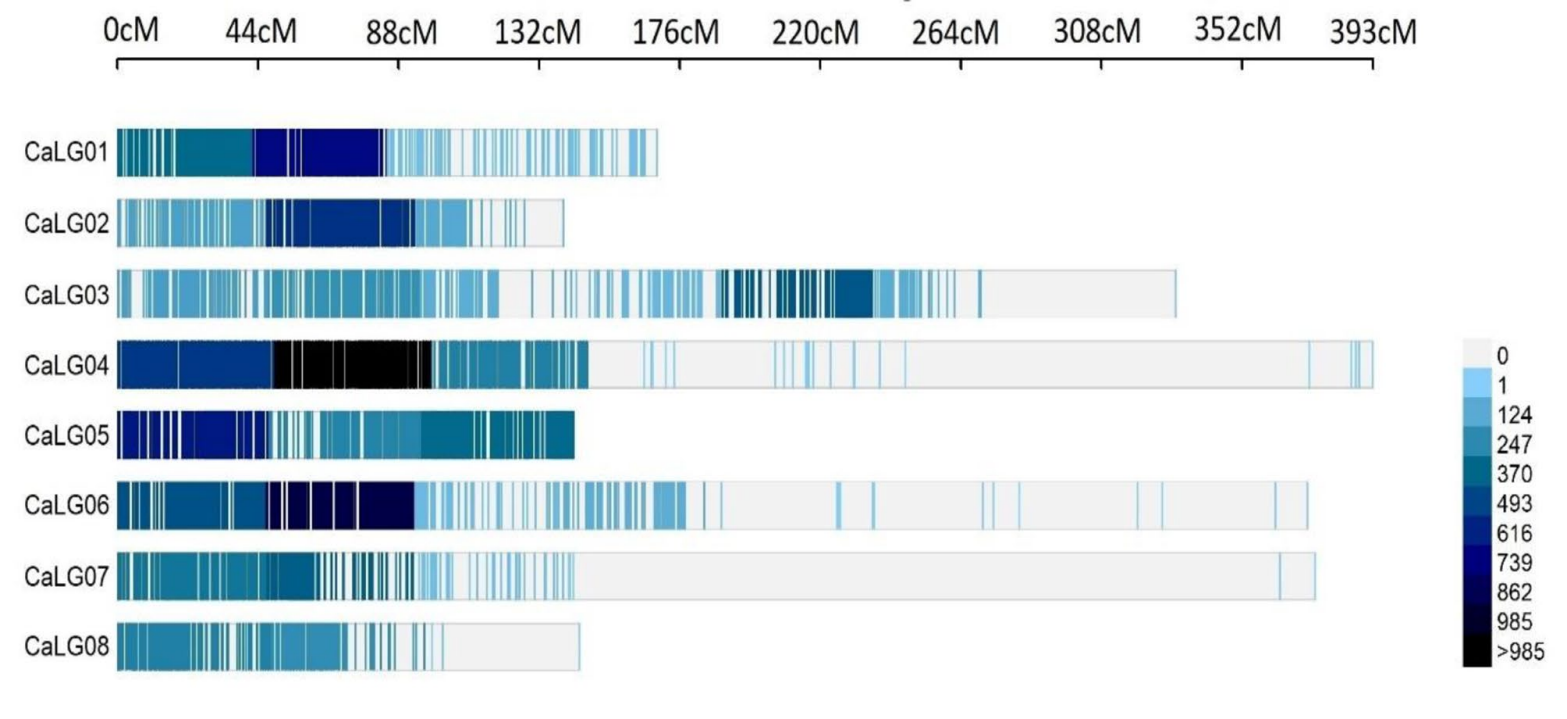
Marker density plot over different chickpea chromosomes. The regions with higher marker density are shown in deeper colour

The high-density consensus map “CaConsensusMap_2022” encompassing all 8 chromosomes of chickpea was prepared for the present study. The consensus map showed huge variation in length of the individual linkage groups as well their marker density. The genetic length of the chromosomes varied from 139.64 cM (Chromosome 2) to 392.7 cM (Chromosome 4). The number of molecular markers mapped on each of the linkage groups ranged from 529 in case of chromosome 8 to 1954 in case of chromosome 4 (Fig. 2a). The total map length for all the chromosomes covered by the consensus map was 2066.75 cM on which 9277 markers were mapped with an average density of 5.05 markers per cM (Table 2; Fig. 3). Chromosome 5 showed the highest marker density of 8.94 markers per cM while lowest marker density was observed for chromosome 7 (2.1 markers per cM). The marker density over each linkage group was not uniform. There were more markers clustered towards one end of the linkage group and one end of the centromere as different types of markers were used to construct the consensus map. A total of 1189 QTL were projected onto the newly prepared consensus map while rest QTL could not be projected due to various reasons such as low AIC values.

**Table 2:** Distribution of markers on “CaConsensusMap_2022”

Linkage Group	No. of markers	Size	Marker Density
CaLG01	1186	168.82	7.03
CaLG02	858	139.64	6.14
CaLG03	1138	331.1	3.44
CaLG04	1954	392.7	4.98
CaLG05	1276	142.795	8.94
CaLG06	1550	372.26	4.16
CaLG07	786	374.72	2.10
CaLG08	529	144.71	3.66
Total	9277	2066.75	(Average) 5.05

### MQTL analysis for abiotic stress tolerance

**Fig. 4:**
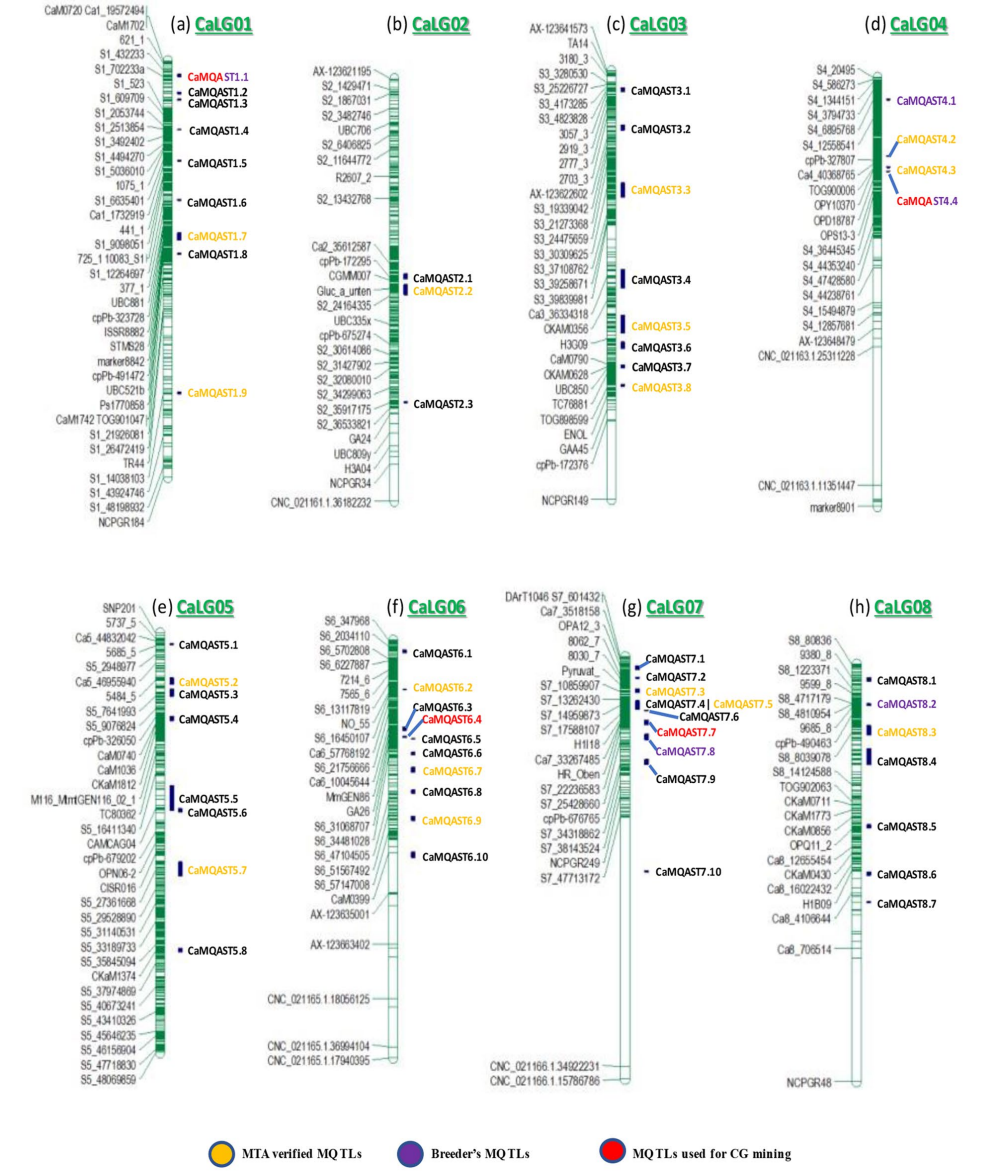
(**a**-**h**): Distribution of MQTL conferring abiotic stress tolerance over the prepared consensus genetic map of chickpea. CaMQAST1.1 and CaMQAST4.4 have been shown in mixed color as they have been taken for CG mining as well as recommended for breeding purposes

**Fig. 5:**
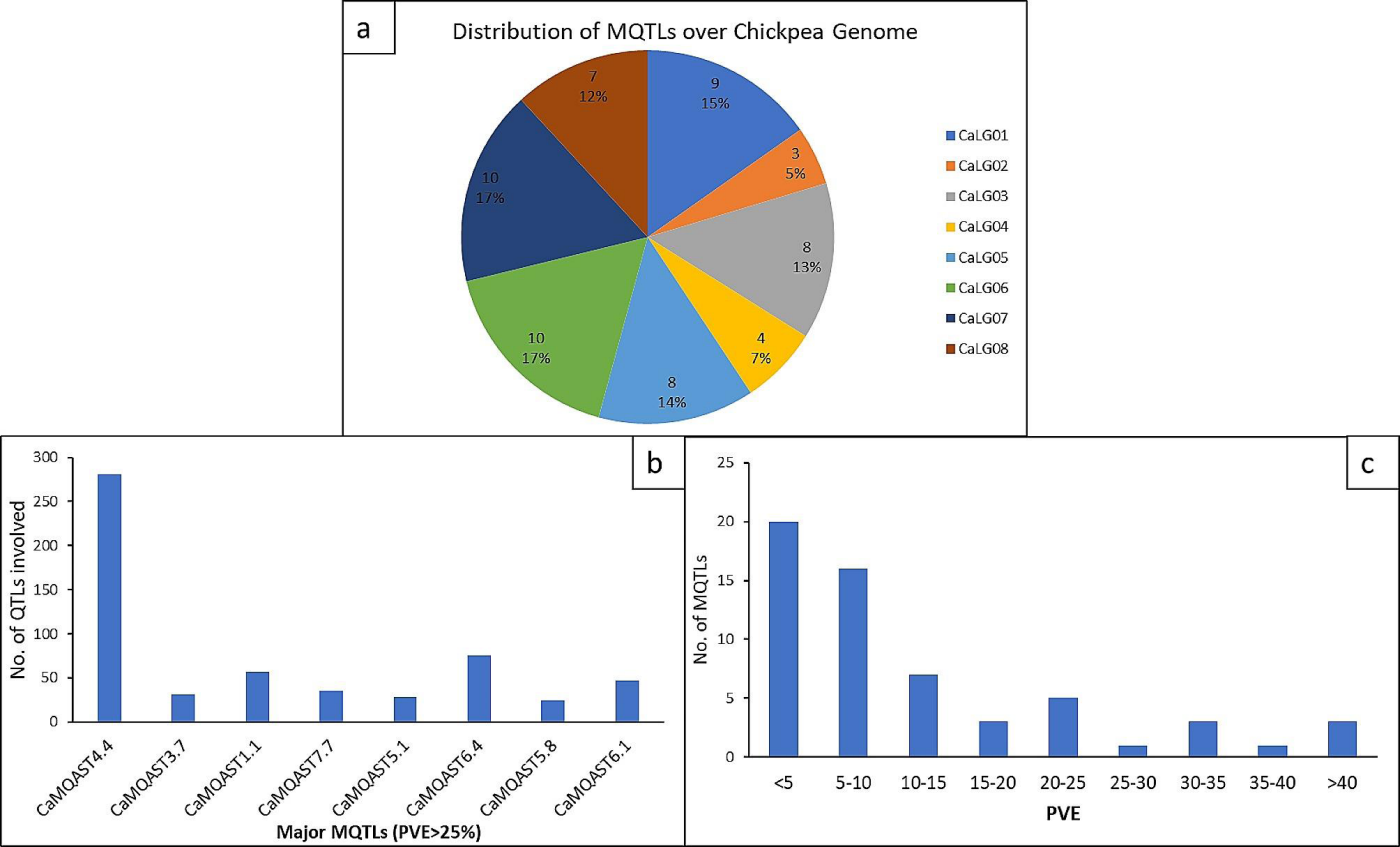
Characteristics of MQTL related to abiotic stress tolerance as obtained in the present study. Distribution of MQTL related to abiotic stress tolerance in chickpea genome (**a**), number of QTL involved in major MQTL (PVE > 25) (**b**), Distribution of MQTL according to PVE (**c**)

The approach given by [99] was used to predict the MQTL on the chickpea genome as all of the chromosomes had more than 10 reported QTL. In this study, a total of 59 MQTL were predicted for a collective tolerance towards heat, cold, drought, and salinity stress from a total of 1158 QTL (Table 3). The MQTL were distributed over the 8 chromosomes. The highest number of MQTL were reported on Chromosome 6 and 7 with 10 MQTL each, together amounting to more than one third of the total MQTL while the least number of MQTL were reported on Chromosome 2 (3 MQTL) contributing only 5% of the total MQTL (Fig. 4). The chromosome 1 consisted of 9 MQTL followed by chromosomes 3 and 5 which have 8 MQTL each. This was followed by Chromosome 8 having 7 MQTL and chromosome 4 having 4 MQTL. Two MQTL were found to overlap with each other on chromosome 5 (viz. CaMQAST5.5 and CaMQAST5.6). The PVE by the MQTL ranged from 1% in a few minor MQTL such as CaMQAST1.3, CaMQAST6.2, CaMQAST6.3, etc. to 66% in one of the major MQTL, viz. CaMQAST4.4. The characteristics of MQTL reported are represented in Fig. 5. Maximum number of MQTL had < 10% PVE, the number decreasing with every 10% increase in PVE until only 3 MQTL were found to have > 40% of PVE (Fig. 5c). These 3 MQTL were considered to be highly significant in contributing tolerance towards heat, cold, drought, and salinity stress (PVE ≥ 40%). On average, the MQTL had 12.136% PVE and 2.469 CI (95%).

**Table 3:** Details of MQTL obtained from BioMercator v4.2.3

S. no.	MQTL	Linkage Group	Position	CI (95%)	*R* ^2^	Marker Interval
1	CaMQAST1.1	CaLG01	5.87 (5.47–6.27)	0.8	42	S1_432233-S1_702233
2	CaMQAST1.2	CaLG01	13.02 (12.695–13.345)	0.65	8	SNP5-AX-123,644,833
3	CaMQAST1.3	CaLG01	15.83 (15.695–15.965)	0.27	1	DArT1786-DArT1798
4	CaMQAST1.4	CaLG01	27.9 (27.76–28.04)	0.28	5	S1_2513914-S1_2649906
5	CaMQAST1.5	CaLG01	40.6 (40.52–40.68)	0.16	5	1120_1-S1_6880666
6	CaMQAST1.6	CaLG01	56.32 (56.175–56.465)	0.29	2	TA116-TA43
7	CaMQAST1.7	CaLG01	71.25 (69.875–72.625)	2.75	6	S1_16552817-S1_17492638
8	CaMQAST1.8	CaLG01	78.3 (78.23–78.37)	0.14	23	H5A08-CKaM1904
9	CaMQAST1.9	CaLG01	134.68 (134.45-134.91)	0.46	7	S1_14038103-S1_13991104
10	CaMQAST2.1	CaLG02	65.31 (64.57–66.05)	1.48	6	OPZ03943-M10_MtmtGEN10_03_1
11	CaMQAST2.2	CaLG02	69.66 (68.07–71.25)	3.18	14	CKaM1101-S2_24093127
12	CaMQAST2.3	CaLG02	106.9 (106.835-106.965)	0.13	18	S2_35795497-S2_35875671
13	CaMQAST3.1	CaLG03	14.35 (12.955–15.745)	2.79	7	3113_3-3068_3
14	CaMQAST3.2	CaLG03	43.69 (42-45.38)	3.38	12	2584_3-S3_15583265
15	CaMQAST3.3	CaLG03	92.01 (86.72–97.3)	10.58	7	S3_28996181-S3_32662861
16	CaMQAST3.4	CaLG03	160.5 (153.37-167.63)	14.26	2	AX-123,621,900-AX-123,621,911
17	CaMQAST3.5	CaLG03	195.61 (188.89-202.33)	13.44	2	CKAM0554-Ca3_22008087
18	CaMQAST3.6	CaLG03	212.07 (209.655-214.485)	4.83	4	SCAF10_699507-Ca3_31267675
19	CaMQAST3.7	CaLG03	228.43 (227.425-229.435)	2.01	56	NCPGR12-SCAF8210_1451
20	CaMQAST3.8	CaLG03	242.74 (242.055-243.425)	1.37	7	CaM0610-TOG912320
21	CaMQAST4.1	CaLG04	20.09 (19.755–20.425)	0.67	15	S4_6613796-S4_6841834
22	CaMQAST4.2	CaLG04	71.9 (71.845–71.955)	0.11	5	H5A04-S4_24013381
23	CaMQAST4.3	CaLG04	82.05 (81.89–82.21)	0.32	8	Ca4_38370902-Ca4_37349212
24	CaMQAST4.4	CaLG04	85.96 (85.92-86)	0.8	66	TOG900323-TOG906662
25	CaMQAST5.1	CaLG05	3.71 (3.66–3.76)	0.1	33	S5_31260243-5690_5
26	CaMQAST5.2	CaLG05	16.36 (15.28–17.44)	2.16	5	S5_5404385-S5_6300360
27	CaMQAST5.3	CaLG05	20.31 (19.115–21.505)	2.39	2	cpPb-682328-5426_5
28	CaMQAST5.4	CaLG05	29.02 (28.35–29.69)	1.34	21	cpPb-326,684-S5_10406613
29	CaMQAST5.5	CaLG05	56.21 (52.015–60.405)	8.39	2	S5_40439514-AX-123,631,517
30	CaMQAST5.6	CaLG05	60.32 (59.87–60.77)	0.9	3	CAMCAG04-AX-123,631,517
31	CaMQAST5.7	CaLG05	80.3 (78.03–82.57)	4.54	2	S5_26477751-S5_28239535
32	CaMQAST5.8	CaLG05	107.96 (107.4-108.52)	1.12	32	S5_36251841-S5_36672164
33	CaMQAST6.1	CaLG06	9.94 (9.005–10.875)	1.87	29	7704_6-S6_3985519
34	CaMQAST6.2	CaLG06	43.68 (43.66–43.7)	0.04	1	AX-123,642,585-AX-123,663,334
35	CaMQAST6.3	CaLG06	78.9 (77.635–80.165)	2.53	1	SCAF11_4875713-OPC06_1
36	CaMQAST6.4	CaLG06	86.01 (85.465–86.555)	1.09	33	CKaM1351-Ca6_2214178
37	CaMQAST6.5	CaLG06	87.96 (87.905–88.015)	0.11	13	AX-123,634,395-AGL76
38	CaMQAST6.6	CaLG06	101.12 (100.02-102.22)	2.2	1	S6_33334977-S6_34480990
39	CaMQAST6.7	CaLG06	115.52 (113.61-117.43)	3.82	2	S6_37280189-S6_39781068
40	CaMQAST6.8	CaLG06	134.9 (133.63-136.17)	2.54	7	Pgd1-S6_45695043
41	CaMQAST6.9	CaLG06	158.75 (156.99-160.51)	3.52	1	S6_52417747-S6_53683753
42	CaMQAST6.10	CaLG06	191.38 (189.12-193.64)	4.52	1	CaM0399-AX-123,640,392
43	CaMQAST7.1	CaLG07	9.67 (8.435–10.905)	2.47	4	8180_7-S7_4411515
44	CaMQAST7.2	CaLG07	18.47 (18.07–18.87)	0.8	14	8045_7-S7_6970143
45	CaMQAST7.3	CaLG07	30.02 (28.425–31.615)	3.19	5	S7_10050020-S7_11192667
46	CaMQAST7.4	CaLG07	40.77 (39.07–42.47)	3.4	13	CAMCAG01-Ca7_30026017
47	CaMQAST7.5	CaLG07	44.84 (43.555–46.125)	2.57	5	S7_15099099-S7_16104217
48	CaMQAST7.6	CaLG07	47.69 (47.61–47.77)	0.16	2	S7_16381934-S7_16626703
49	CaMQAST7.7	CaLG07	58.07 (56.305–59.835)	3.53	35	cpPb-682,222-S7_20579963
50	CaMQAST7.8	CaLG07	71.07 (68.945–73.195)	4.25	11	S7_23439225-S7_25218332
51	CaMQAST7.9	CaLG07	93.21 (91.125–95.295)	4.17	4	ICCeM0033-CKAM1317
52	CaMQAST7.10	CaLG07	190.24 (189.855-190.625)	0.77	6	S7_48148505-CNC_021166.1.34922231
53	CaMQAST8.1	CaLG08	5.24 (4.695–5.785)	1.09	18	9385_8-9416_8
54	CaMQAST8.2	CaLG08	13.94 (13.685–14.195)	0.51	22	S8_4717179-S8_4980923
55	CaMQAST8.3	CaLG08	22.83 (21.31–24.35)	3.04	4	S8_7193960-S8_8293716
56	CaMQAST8.4	CaLG08	31.98 (29.325–34.635)	5.31	1	TOG895142-S8_3639513
57	CaMQAST8.5	CaLG08	56.21 (55.68–56.74)	1.06	11	ICCM0130a-S8_7118591
58	CaMQAST8.6	CaLG08	72.6 (72.12–73.08)	0.96	22	CKaM1750-ICCeM054
59	CaMQAST8.7	CaLG08	82.44 (82.4-82.48)	0.08	22	Ca8_4106644-Ca8_3050452

Of the 1189 QTL projected, 1158 QTL were predicted for MQTL analysis while the other 29 QTL were not predicted to be involved in any MQTL by the software. Surprisingly, the major MQTL with the highest PVE also had the most QTL involved. The MQTL CaMQAST4.4 involved the highest number of QTL (281) of which 95 QTL were specific to itself while 144 QTL were shared between CaMQAST4.3 and CaMQAST4.4, and 42 QTL were shared between CaMQAST4.4, CaMQAST4.3, and CaMQAST4.2. In total, 628 QTL were specifically contributing to a single MQTL while 410 QTL were shared between 2 MQTL, 100 QTL were involved in 3 MQTL, 15 QTL were involved in 4 MQTL each, 3 QTL involved in 5 MQTL each and one QTL (QR9100SDWT2) was contributing towards 6 MQTL, viz., CaMQAST5.1, CaMQAST5.2, CaMQAST5.3, CaMQAST5.4, CaMQAST5.5 and CaMQAST5.6 (Supplementary Table 2).

Fifteen MQTL were found to exclusively consist of QTL reported for drought tolerance while 5 MQTL were found to consist of salinity tolerance conferring QTL (Table 4). The MQTL exclusively involved in inducing heat or cold tolerance was not observed. CaMQAST3.8, CaMQAST4.2, and CaMQAST4.4 were involved in conferring all 4 kinds of stress tolerance to the plants, indicating the presence of a common pathway for inducing stress tolerance.

**Table 4:** Contribution of MQTL towards the type of stress tolerance

S. no.	MQTL ID	Contribution to Drought tolerance (%)	Contribution to Heat tolerance (%)	Contribution to Salinity tolerance (%)	Contribution to Cold tolerance (%)	Traits associated with MQTL
1	CaMQAST1.1	86.0	10.5	3.5	-	NSB, YLD, 100SW, NPB, DF, HI, DM, BM, PS, SN, DTI, VS, %PS
2	CaMQAST1.2	64.0	20.0	16.0	-	100SW, VS, DTI, %PS, YLD, BM, PHT, AGR30, AGR34, HI
3	CaMQAST1.3	45.5	18.2	36.4	-	VS, YLD, 100SW, AGR30, AGR34, BM, WUE
4	CaMQAST1.4	10.0	50.0	40.0	-	BM, VS, WUE, DTI, YLD, HI, DM
5	CaMQAST1.5	12.5	50.0	37.5	-	YLD, HI, VS, DM, SW, SBM
6	CaMQAST1.6**	100.0	-	-	-	SW, DTI, RDW, 100SW
7	CaMQAST1.7	78.6	-	21.4	-	HI, YLD, 100SW, RDW, DSI
8	CaMQAST1.8	90.6	-	9.4	-	100SW, RDW, YLD, DSI, HI, DF, DM, BM, RTR, DFM, DTS, Tc–Ta, PHT, NSB, DTI
9	CaMQAST1.9**	100.0	-	-	-	100SW, YLD, DTI, BM
10	CaMQAST2.1**	-	-	100.0	-	YLD
11	CaMQAST2.2**	100.0	-	-	-	YLD
12	CaMQAST2.3**	100.0	-	-	-	RLWC, MSI
13	CaMQAST3.1	25.0	-	75.0	-	NPB, YLD, DM, DF
14	CaMQAST3.2	33.3	-	55.6	11.1	DF, HI, SW, CT, YLD, DM
15	CaMQAST3.3**	-	-	100.0	-	YLD, DM, N, 100SW
16	CaMQAST3.4**	-	-	100.0	-	100SW
17	CaMQAST3.5**	100.0	-	-	-	DF, PHT
18	CaMQAST3.6**	100.0	-	-	-	DM, YLD, PHT
19	CaMQAST3.7	96.8	-	-	3.2	PHT, DM, Tc–Ta, YLD, HI, BM, RDW, NP/P, DF, RDp, NS/P, 100SW, DFM, DTS, VR
20	CaMQAST3.8*	50.0	16.7	16.7	16.7	PHT, VR, SC, BM, NPB
21	CaMQAST4.1	78.3	-	21.7	-	100SW, DM, NP/P, Δ13c, YLD, RTR, SN, PHT, HI, DF, NFP, PWDBM, DSI, N, WUE
22	CaMQAST4.2*	74.1	3.4	19.0	3.4	BM, 100SW, HI, NFP, Cl-, SBM, WUE, SS, SW, SN, N, PHT, RLD, NP/P, Δ13c, DTI, RSA, DM, DF
23	CaMQAST4.3	99.5	-	-	0.5	PHT, BM, 100SW, NS/P, NP/P, RTR, DSI, HI, DF, RLD, Δ13c, DM, NSB, 3DL, PLA, PBS, PHTG, DTI, RSA, SLW, SLA, LAI, 3DLG, eTR, TR, T, R-3D/PLA, eT, YLD, SDW
24	CaMQAST4.4*	96.1	1.1	2.1	0.7	PHT, NP/P, RLD, HI, BM, 100SW, Δ13c, DM, NSB, RTR, 3DL, PLA, PBS, PHTG, DTI, RSA, DF, SLW, SLA, LAI, 3DLG, eTR, TR, T, R-3D/PLA, eT, YLD, DSI, SDW, DFI, SN, Tc–Ta, AGR34, DTF, %PS, NFP, AGR30, VS, NS/P
25	CaMQAST5.1	89.3	-	10.7	-	RLWC, PHT, DF, DM, RLD, RSA, YLD, RDp, PS, 100SW
26	CaMQAST5.2	81.8	-	18.2	-	DF, DM, RLD, RSA, YLD, RDp, PS, PWDPHT, 100SW, HI, NS/P
27	CaMQAST5.3	58.8	-	41.2	-	DF, YLD, PWDPHT, 100SW, DM, HI, NS/P
28	CaMQAST5.4	44.4	11.1	44.4	-	YLD, DF, 100SW, DM, HI, PHT, NS/P, BM, SN, NFP, Na
29	CaMQAST5.5	50.0	-	50.0	-	100SW, SW, DF
30	CaMQAST5.6	40.0	-	60.0	-	100SW, SW, PHT, DF
31	CaMQAST5.7	50.0	-	50.0	-	DF, Cl-
32	CaMQAST5.8**	-	-	100.0	-	BM, YLD, 100SW, HI, DM, ADM, N
33	CaMQAST6.1	51.1	-	48.9	-	100SW, SN, YLD, HI, DF, DM, PHT, DTI, NP/P, PS, MPI, BM
34	CaMQAST6.2**	-	-	100.0	-	NP/P, 100SW
35	CaMQAST6.3**	100.0	-	-	-	YLD, PHT, RLD, NPB, RV, RSA, SDW, HI, DF, DM, T, R-3D/PLA, 100SW, 3DLG
36	CaMQAST6.4**	100.0	-	-	-	PHT, RTR, RLD, NPB, RV, RSA, SDW, YLD, HI, R-3D/PLA, DF, Tc–Ta, DM, T, 100SW, 3DLG, PHTG, SLA, NP/P, TR, BM
37	CaMQAST6.5	98.6	-	1.4	-	RTR, PHT, RLD, NPB, RV, RSA, SDW, YLD, HI, R-3D/PLA, DF, DM, T, 100SW, 3DLG, PHTG, SLA, NP/P, TR, BM, Tc–Ta
38	CaMQAST6.6	92.3	7.7	-	-	NPB, RV, RSA, SDW, PHT, R-3D/PLA, DM, MSI, %PS
39	CaMQAST6.7	25.0	50.0	25.0	-	NPB, SN, YLD
40	CaMQAST6.8	66.7	25.0	8.3	-	DM, PHT, HI, VS, CC, YLD
41	CaMQAST6.9**	100.0	-	-	-	HI, SW, PHT
42	CaMQAST6.10	50.0	-	50.0	-	DF, YLD
43	CaMQAST7.1	20.0	-	80.0	-	RLWC, YLD, 100SW, BM
44	CaMQAST7.2	6.3	-	93.8	-	YLD, 100SW, BM, TPN, HI, NP/P, SN, DM
45	CaMQAST7.3	62.5	-	37.5	-	DM, MSI, PHT, R-3D/PLA, TPN, YLD, SN
46	CaMQAST7.4	64.7	-	35.3	-	R-3D/PLA, TPN, YLD, SN, PHT, DM, HI, DTI, BM
47	CaMQAST7.5	53.3	-	46.7	-	TPN, YLD, SN, PHT, DM, HI, R-3D/PLA, NFP, DTI, BM
48	CaMQAST7.6	69.2	-	30.8	-	TPN, YLD, SN, R-3D/PLA, PHT, DTI, 100SW
49	CaMQAST7.7**	100.0	-	-	-	R-3D/PLA, PHT, DSI, DTS, DM, PHTG, eTR, DFM, SC, TR, RDW, YLD, BM, RLD, RV
50	CaMQAST7.8**	100.0	-	-	-	PHT, RDW, YLD, BM, RLD, DSI, RV, SW, NS/P, DM, DF
51	CaMQAST7.9	60.0	-	40.0	-	BM, NPB, DTI, SN, NFP
52	CaMQAST7.10**	100.0	-	-	-	BM, NP/P, MPI, 100SW, YLD
53	CaMQAST8.1	94.9	3.8	-	1.3	HI, VS, %PS, PWDDF, YLD, DSI, DM, 100SW, RTR, BM, RDp, DTI, NPB, PHT, PS, NP/P, RDW, CT
54	CaMQAST8.2	97.2	-	0.9	1.8	DM, 100SW, RTR, DF, BM, HI, RDp, DTI, NPB, YLD, PHT, PS, NP/P, RDW, CT
55	CaMQAST8.3**	100.0	-	-	-	PHT, HI, 100SW, DM, DF
56	CaMQAST8.4**	100.0	-	-	-	DF, DM
57	CaMQAST8.5	72.7	-	24.2	3.0	DM, DF, RSA, PHT, SN, RV, HI, BM
58	CaMQAST8.6**	100.0	-	-	-	PHTG, NS/P, DM, NPB, PHT, DTS, DF, BM, HI, 3DL, 100SW, RTR, DTI, LAI, DSI, NSB, T
59	CaMQAST8.7	98.9	-	1.1	-	DM, DF, NPB, DSI, HI, PHT, BM, NSB, T, YLD

* MQTL contributing for all of the mentioned abiotic stress tolerance, viz. drought, heat, cold, and salinity

** MQTL contributing for only one kind of abiotic stress tolerance

### MQTL validated with previously reported GWA studies

**Fig. 6:**
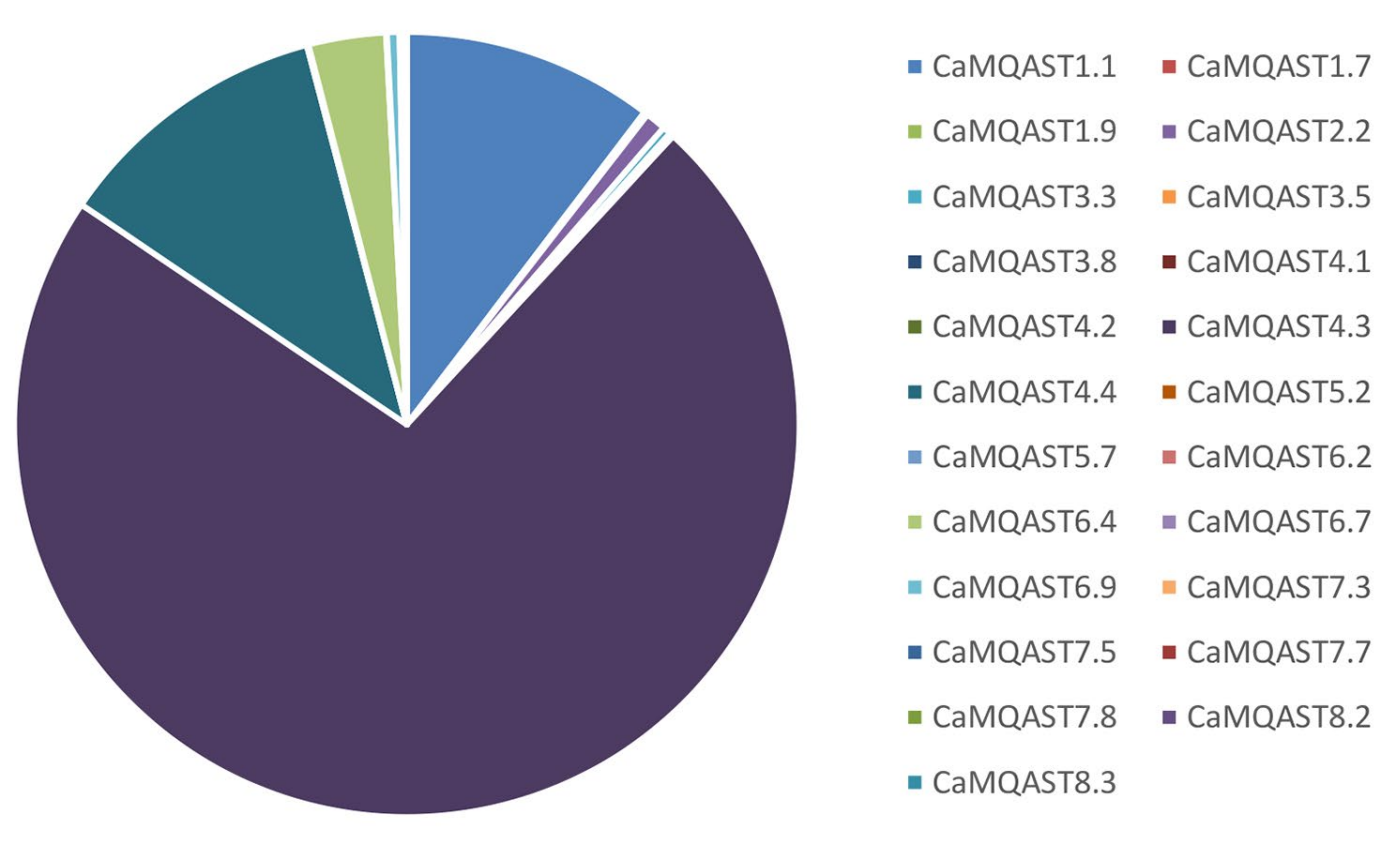
Frequencies of known MTAs co-located with the 23 MQTL validated in this study

The physical locations of the nearest flanking markers for each MQTL were obtained by reviewing literatures or by performing BLASTn of the primers or marker sequence against chickpea genome. Out of the 59 MQTL reported in this study, the physical location of one or both of the flanking markers in 16 MQTL could not be obtained as they were either flanked by AFLP, RFLP, etc. or other markers whose sequence information could not be obtained. The physical intervals of the MQTL ranged from 46 Kb (CaMQAST1.9) to ∼ 19.5 Mb (CaMQAST7.7). Many MQTL were found to be co-located with different stress tolerance associated regions of the genome reported by multiple studies. The number of MTAs co-located with each MQTL also varied. A total of 23 MQTL were found to cover the physical locations associated with traits related to abiotic stress tolerance as reported by different GWA studies (Table 5; Fig. 6). All the MQTL reported on chromosome 4 were found to overlap with reported MTAs.

**Table 5:** List of MQTL verified with Marker Trait Association studies

S. no.	QTL	Linkage Group	Position (cM)	*R* ^2^	Start bp	Stop bp	Interval (bp)	Genome-wide Association Studies
1	CaMQAST1.1^#+^	CaLG01	5.87	42	4,32,233	7,02,233	**2,70,000**	[104, 107, 108]
2	CaMQAST1.7	CaLG01	71.25	6	1,65,52,817	1,74,92,638	9,39,821	[107]
3	CaMQAST1.9	CaLG01	134.68	7	1,40,38,103	1,39,91,104	46,999	[109]
4	CaMQAST2.2	CaLG02	69.66	14	3,03,46,113	2,40,93,127	62,52,986	[101, 104, 107–109]
5	CaMQAST3.3	CaLG03	92.01	7	2,89,96,181	3,26,62,861	36,66,680	[108, 109]
6	CaMQAST3.5	CaLG03	195.61	2	2,84,59,930	2,20,08,087	64,51,843	[108]
7	CaMQAST3.8	CaLG03	242.74	7	3,27,48,128	3,26,61,055	87,073	[108]
8	CaMQAST4.1^+^	CaLG04	20.09	15	66,13,796	68,41,834	2,28,038	[107–109]
9	CaMQAST4.2	CaLG04	71.9	5	2,86,36,088	2,40,13,381	46,22,707	[104, 107, 108]
10	CaMQAST4.3	CaLG04	82.05	8	3,83,70,902	3,73,49,212	10,21,690	[107–109]
11	CaMQAST4.4^#+^	CaLG04	85.96	66	3,89,60,478	4,03,58,494	**13,98,016**	[107–109]
12	CaMQAST5.2	CaLG05	16.36	5	54,04,385	63,00,360	8,95,975	[108]
13	CaMQAST5.7	CaLG05	80.3	2	2,64,77,751	2,82,39,535	17,61,784	[107, 108]
14	CaMQAST6.2	CaLG06	43.68	1	5,76,32,163	5,77,61,758	1,29,595	[107]
15	CaMQAST6.4^#^	CaLG06	86.01	33	87,06,550	22,14,178	**64,92,372**	[108]
16	CaMQAST6.7	CaLG06	115.52	2	3,72,80,189	3,97,81,068	25,00,879	[101, 107, 109]
17	CaMQAST6.9	CaLG06	158.75	1	5,24,17,747	5,36,83,753	12,66,006	[107, 108]
18	CaMQAST7.3	CaLG07	30.02	5	1,00,50,020	1,11,92,667	11,42,647	[107–109]
19	CaMQAST7.5	CaLG07	44.84	5	1,50,99,099	1,61,04,217	10,05,118	[108]
20	CaMQAST7.7^#^	CaLG07	58.07	35	10,86,709	2,05,79,963	**1,94,93,254**	[104, 105]
21	CaMQAST7.8^+^	CaLG07	71.07	11	2,34,39,225	2,52,18,332	17,79,107	[107, 108]
22	CaMQAST8.2^+^	CaLG08	13.94	22	47,17,179	49,80,923	2,63,744	[108]
23	CaMQAST8.3	CaLG08	22.83	4	71,93,960	82,93,716	10,99,756	[108]

^#^Major MQTL (PVE > 30%) taken for candidate gene mining

^+^Promising MQTL recommended for breeding approaches

### Candidate genes underlying MQTL

A majority of the MQTL validated via MTA studies had very low PVE. Out of the 23 MQTL, only 4 MQTL had PVE > 30%, one MQTL had 22% PVE, 3 MQTL had PVE < 20% and the rest (18) MQTL had PVE < 10%. Only the major MQTL having PVE > 30% were mined for candidate genes conferring abiotic stress tolerance in chickpea as they were considered to be significantly involved in imparting abiotic stress tolerance in plants. The genes whose annotations were not found or uncharacterized were not considered as their function could not be determined. CaMQAST6.4 covered the maximum number of genes (158) followed by CaMQAST7.7 (57 genes), CaMQAST1.1 (32 genes), and CaMQAST4.4 (29 genes). The putative candidate genes which were found to be directly involved in imparting abiotic stress tolerance in chickpea were marked (Supplementary Table 3). Many genes were found to be present in multiple copies. These include F-box/kelch-repeat proteins, aspartic protease, ubiquitin-protein ligase RING-like protein, cytochrome P450, transcription factors, cell cycle control proteins, pentatricopeptide repeat-containing protein, serine/threonine-protein, phosphatase 7 protein, filament-like plant protein, UDP-glycosyltransferases, and zinc finger domain proteins. Apart from these genes, some genes which might involve in stress tolerance-inducing pathways were also found. These included genes responsible for hormonal activity such as gibberellin 20 oxidase, ABA-insensitive 5-like protein, ABA hydroxylase 4-like protein, and IAA-amino acid hydrolase, membrane transporters such as Casparian strip membrane proteins, potassium channel proteins, aquaporins, and vacuolar amino acid transporter, and other proteins such as root meristem growth factor, peroxidase, cinnamoyl-CoA reductase, heat shock proteins (hsp), chaperone dnaJ-like protein, and trichome birefringence-like proteins. The unpredicted proteins may be taken for further studies so as to learn their functions regarding stress response.

## Discussion

The genetics of quantitative resistance to abiotic stress has been explored in numerous cultivars, mainly as a result of the discovery of an increased number of molecular markers, the decreased cost of genotyping mapping populations, and improved QTL mapping methodologies [79]. This study analyzed 1512 QTL in chickpea cultivars, focusing on abiotic stress tolerance (Supplementary Table 1). However, some QTL found in one population did not work well in breeding programs with different populations [80]. To better utilize these loci, the study reanalyzed all loci together. Meta-analysis of QTL is a promising method for integrating and predicting stable and robust QTL, addressing heterogeneity between studies [81].

**Fig. 7:**
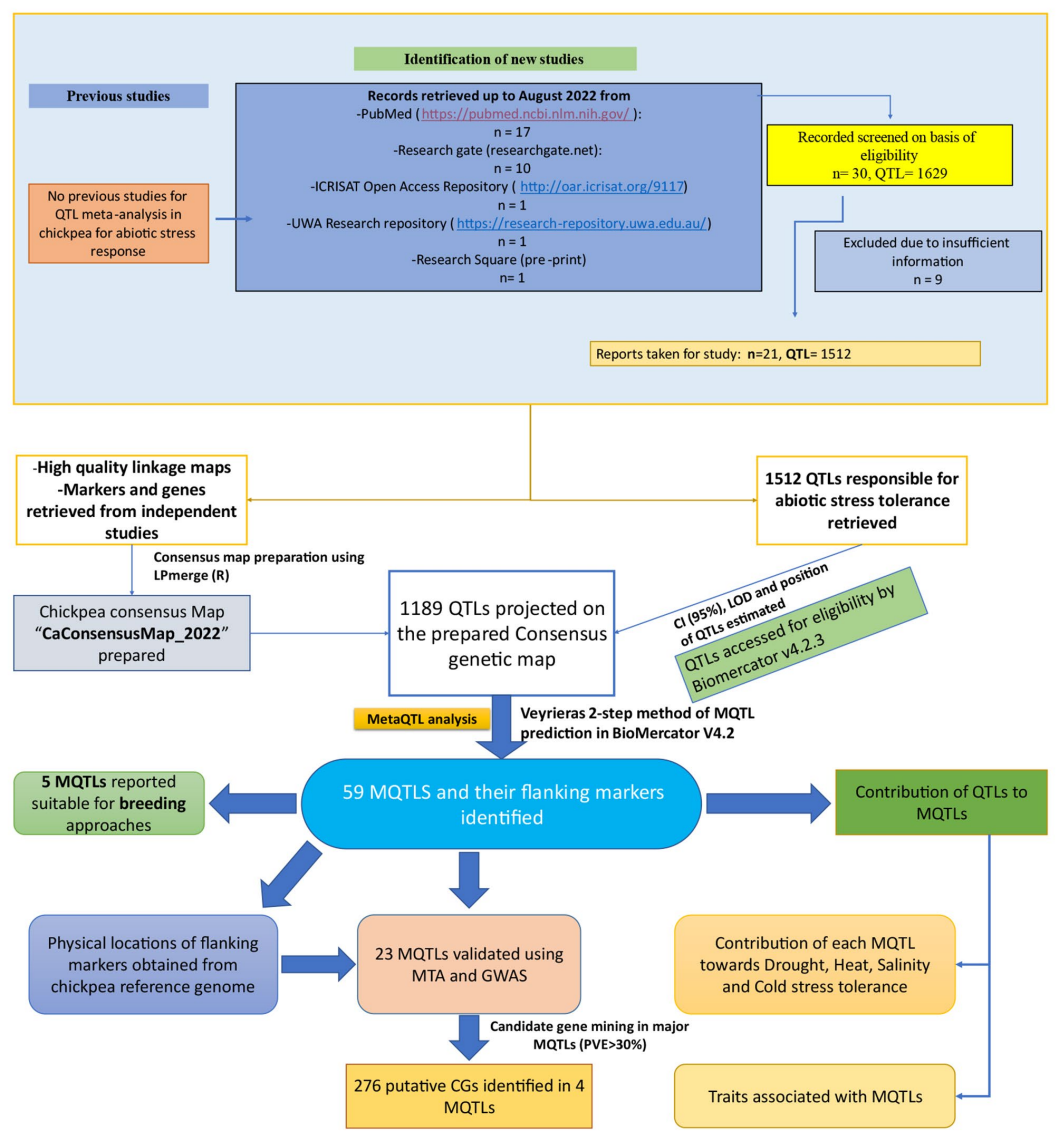
Workflow and results obtained by MQTL analysis in this study

This study aims to compile studies on chickpea breeding programs, focusing on QTLs and markers for individual stress tolerance. The goal is to provide concise and accurate data for chickpea breeders and researchers (Fig. 7).

### Prediction of MQTL and their validation using MTA studies

In the present study, a total of 1512 QTL, 1189 of which were projected on the prepared chickpea consensus map CaConsensusMap_2022, out of which 1158 QTL were used to predict 59 MQTL. The MQTL were found to be mostly distributed towards one side of the centromere, generally towards the high marker dense regions. It is assumed that QTL density is dependent upon high gene density and rate of polymorphism [82]. Similar observations have been made in common bean and soybean where the QTL and MQTL were found to be distributed towards sub-telomeric and centromeric regions of high marker density [75, 76]. Most of the existing QTL studies were related to drought tolerance in chickpea as this is the most prevalent and well-studied stress in major chickpea-growing countries (ICRISAT).

Around 39% of MQTL contributed > 10% to abiotic stress tolerance, while most contribute a small portion. This contrasts with the predicted QTL characteristics, where over 61% contribute > 10%. Stress tolerance is governed by multiple loci with minor effects [73], with many main effect QTL overlapping and grouped under a small number of QTL.

Chromosome 4 has been reported to be actively involved in conferring different abiotic stress tolerances such as drought and salinity tolerance [78, 83] while chromosomes 3 and 6 have been reported to harbour QTL conferring heat tolerance [69]. The MQTL responsible for the highest PVE, CaMQAST4.4 (66%) was also located on chromosome 4. It is also involved in providing tolerance to drought, heat, cold, and salinity stresses. About 39% of the predicted MQTL were validated using GWA studies. This low percentage was mainly due to the unavailability of physical location or sequence information of the flanking markers in the consensus map. The details of physical locations and markers obtained from GWAS have been provided in Table 5. Among these validated MQTL, those having smaller intervals (< 2 Mb), high PVE (> 10%) and including a large number of originally reported QTL were recommended to be used for breeding programs [75]. Five MQTL CaMQAST1.1, CaMQAST4.1, CaMQAST4.4, CaMQAST7.8, and CaMQAST8.2 were selected as breeder’s QTL as they have the desirable qualities which can be used for breeding approaches.

It was observed that MQTL conferring heat or cold stress tolerance were involved in inducing tolerance to drought, salinity, or both stresses, but not vice versa. This may be due to different stress-related genes on the same genomic region and the activation of the same mechanism in drought and salinity tolerance. To observe the genetic relationships between various stress responses in chickpea, further studies are necessary. From a physiological point of view, heat and cold stress lead to damage in membranes which induces an ion imbalance [38, 84] and the same results can be observed during drought and salinity stress.

### Candidate genes mined from MQTL regions

In the regions covered by the MQTL, many proteins belonging to protein superfamilies such as kinase-like domain superfamily, F-box-like domain superfamily, UDP glucuronosyl/ UDP-glucosyltransferase, zinc finger proteins (RING/FYVE/PHD-type), etc. were present, which confirm to the reports provided in other MQTL studies [67, 73] Different putative CGs were marked for regions covered by each of the MQTL which might be involved in abiotic stress resistance pathways in plants. Many genes involved in protein modification activity, such as aspartic proteases, ubiquitin-protein ligases, etc., were present in the MQTL regions.

In the genomic region covered by CaMQAST1.1, two transport proteins- Ca6885 and Ca6325 code for potassium channel protein and bidirectional sugar transporter respectively, a gene coding for elongation of fatty acids A (Ca6828), suppressor of gene silencing 3 like protein (Ca5169), and protein-coding for gibberellin 20 oxidase (Ca7534) were found. These proteins indicate the locus’ involvement in stress tolerance Elongation of fatty acids improves membrane fluidity, while gibberellins trigger osmotic stress response [85]. The presence of transporter proteins indicates that it is responsible for maintaining osmotic potential in cells, which is an important aspect of drought and salinity stress tolerance. The suppressor of gene silencing 3 is important in providing resistance to plants against viral pathogens [86]. Its degradation by a Heat Shock Transcription Factor A2 (HSFA2) and H3K27me3 demethylase Relative to Early Flowering 6 (REF6) feedback loop induces a quick response to heat and heritable trans-generational memory of acclimation to heat while attenuating immunity [87]. The presence of a similar gene signifies its involvement in viral resistance as well as trans-generational heat stress response memory. This information could aid in studying heat stress tolerance and developing heat stress-tolerant crops.

The MQTL CaMQAST4.4 contained Chaperone dnaJ-like protein, trichome birefringence protein, and Casparian strip membrane proteins 1 and 2. Chaperone dnaJ-like protein in conjunction with HSP 70, is known to be involved in protein folding and biogenesis [88]. The trichome birefringence protein family genes are known to be responsible for binding cell wall components while Casparian strip membrane proteins 1 and 2 are involved in lignin deposition. Therefore CaMQAST4.4 is predicted to play a major role in the drought tolerance mechanism.

The region covered by CaMQAST6.4 contained Class I heat shock proteins, a heat and acid-stable phosphoprotein-like protein, Abscisic acid-Insensitive-5-like protein, and Abscisic acid 8’-hydroxylase 4-like protein. These genes are crucial in heat stress response and normal growth [89], for germination and seedling growth [90], and abiotic stress response [91], requiring understanding their expression patterns to predict their function.

The MQTL CaMQAST7.7 region housed two IAA-amino acid hydrolase ILR1-like proteins, which are known to be involved in auxin signaling activation. Auxins are known to induce abiotic stress tolerance in plants. Genes encoding for peroxidase and cinnamoyl-CoA reductase1-like protein were also present which are known to impart abiotic stress tolerance. Peroxidase deactivates the reactive oxygen species produced during stress and cinnamoyl-CoA reductase is involved in the biosynthesis of lignin, which imparts abiotic stress tolerance in plants. Surprisingly two copies of genes coding for TMV resistance protein N-like protein and TIR-NBS-LRR disease resistance protein were found, which guard the plants against pathogens [92, 93]. Hence this MQTL also covers a region imparting tolerance to biotic stress as well.

## Conclusions

Chickpea is grown in different parts of the world and faces different kinds of abiotic stresses. Although many studies have been carried out to identify key regions in the chickpea genome responsible for inducing abiotic stress tolerance in chickpea, the studies include different environmental conditions, markers and varieties, leading to a difference in the results reported. In the present study, we have tried to compute a consensus genetic map in chickpea consisting of different types of markers and predicted the MQTL regions responsible for tolerance towards drought, heat, cold, and salinity stresses. These regions were verified using GWA studies and even the presence of putative candidate genes responsible for imparting stress resistance in four major MQTL were identified. Furthermore, five MQTL were predicted to be suitable targets for breeding programs. This study will be useful for the researchers working to develop new varieties which are tolerant towards drought, heat, cold, and salinity stresses.

## Materials and methods

### A comprehensive study on QTL for abiotic stress tolerance in chickpea

A literature survey was done to identify QTL in chickpea, focusing on their tolerance to drought, heat, and salinity using Google Scholar, PubMed, Wiley online library, Krishikosh and Thesis repository of the University of Western Australia (research-repository.edu.au). The data about the QTL reported by these publications were obtained from their respective supplementary files, where available or from various other sources such as pulse database (https://www.pulsedb.org/organism/641), legume Information Database (https://www.legumeinfo.org), ICRISAT-cMAP (http://cmap.icrisat.ac.in/cgi-bin/cmap_public/map_set_info?species_acc=4). A total of 21 papers were retrieved, containing1512 QTL. For each of the QTL, information type of mapping population, size of population, name of traits, method of mapping, position of QTL and flanking markers / 95% CI, LOD, and phenotypic variance explained (PVE)/R^2^ were collected. In cases, where the LOD score was not given, it was calculated as likelihood ratio/4.6 or where it was mentioned that a threshold value of 3 was taken, the LOD values were considered 3 for all QTL.

### Construction of high-density consensus genetic map

The R Package ‘LPmerge’ was used to construct a high-density genetic consensus map for chickpea. Input files were prepared from the genetic map provided by [94–97]. Transcript map with 1147 loci along with the maps of markers reported in the studies were taken for MQTL analysis. The list of maps was analyzed using ‘LPmerge’ with a maximum interval of 1:3 for each linkage group. Redundant markers were renamed by adding alphabets at the end of the markers according to their relative positions in their respective maps to prevent errors during the calculation of the consensus map. Some poorly represented maps were removed to improve the accuracy of the consensus maps. The root means square error (RMSE) values were compared for each of the maps concerning the consensus map for each interval, and the consensus maps with the least mean RMSE value were chosen for further analysis.

### Projection of QTL on the consensus map

Genetic map files and QTL files were prepared for each study depicting QTL related to abiotic stress tolerance and were uploaded for QTL projection in BioMercator v4.2. The input files of QTL included the name of QTL, traits, environment, place and year of the experiment, chromosome number, linkage group, peak position and 95% CI (flanking marker position), LOD, and R^2^. When studies with multiple instances of the same QTL were reported in different environments, only QTL which explained the highest phenotypic variance were included for analysis. When CI values were not given, CI values were calculated according to the formula given below [98].

For the F_2:3_ and Backcross populations, CI (95%) = 530/ (R^2^ × N).

For the recombinant inbred lines (RILs), CI (95%) = 163/ (R^2^ × N);

Where *N* = population size, 530 and 163 are the population-specific constants.

### Prediction of MQTL by meta-analysis

Meta-analysis of QTL was done using BioMercator v4.2, for each of the chromosomes individually. The method proposed by [99] was used for meta-QTL analysis. The best model was selected from a list that included Akaike information criterion (AIC), AIC3, corrected AIC, Bayesian information criterion (BIC), and an average weight of evidence (AWE) predicted models. The model was considered the best fit if it possessed the lowest values of the selection criteria in at least three of the models.

### Validation of predicted MQTL by reported marker trait association studies

Genome-wide association studies reporting various markers and genomic locations associated with traits related to different abiotic stress tolerance such as Heat, cold, drought, and salinity were reviewed in detail [20, 100–109]. The physical locations of the markers flanking the MQTL were obtained from these studies. In case, the physical location of the markers was not given, the flanking sequences or primer sequences of the markers were used to perform NCBI-BLASTn against the chickpea genome assembly having assembly ID- GCA_006151565.1 submitted by ICRISAT on the GenBank database. The previously reported physical locations of the markers were also verified on the reference genome assembly in the NCBI database. These locations and markers were matched against the region covered by the predicted MQTL.

### Identification of candidate genes underlying MQTL

The MQTL verified with the reported genome-wide association studies were further analysed to obtain predicted genes between the flanking markers. The physical position of the flanking markers was determined using BLASTn against the chickpea genome available on NCBI. Out of the validated MQTL, only the MQTL having PVE > 30% were selected. The MQTL having flanking marker interval < 2 Mb were directly used for the exploration of genes, while for the MQTL having marker interval > 2 Mb, the 1 Mb regions flanking the peak position were mined for candidate genes (CGs). The peak position was determined by the formula given by [73]. The candidate genes were mined from the chickpea genome annotation Chickpea_Desi_uwaV3.0 (http://www.cicer.info/cgi-bin/gb2/gbrowse/desiUWAV3.0) along with their functional descriptions if provided.

### Electronic supplementary material

Below is the link to the electronic supplementary material.

Supplementary Material 1

## Data Availability

All data generated or analyzed during this study are included in this published article [and its supplementary information files].
